# High-Throughput Screening Identifies MicroRNAs Regulating Human PCSK9 and Hepatic Low-Density Lipoprotein Receptor Expression

**DOI:** 10.3389/fcvm.2021.667298

**Published:** 2021-07-12

**Authors:** Coen van Solingen, Scott R. Oldebeken, Alessandro G. Salerno, Amarylis C. B. A. Wanschel, Kathryn J. Moore

**Affiliations:** ^1^Leon H. Charney Division of Cardiology, Department of Medicine, New York University Cardiovascular Research Center, New York University School of Medicine, New York, NY, United States; ^2^Department of Cell Biology, New York University School of Medicine, New York, NY, United States

**Keywords:** microRNA, LDL receptor, lipoprotein, proprotein convertase subtilisin kexin type 9, hepatocytes

## Abstract

Investigations into the regulatory mechanisms controlling cholesterol homeostasis have proven fruitful in identifying low-density lipoprotein (LDL)-lowering therapies to reduce the risk of atherosclerotic cardiovascular disease. A major advance was the discovery of proprotein convertase subtilisin/kexin type 9 (PCSK9), a secreted protein that binds the LDL receptor (LDLR) on the cell surface and internalizes it for degradation, thereby blunting its ability to take up circulating LDL. The discovery that loss-of-function mutations in *PCSK9* lead to lower plasma levels of LDL cholesterol and protection from cardiovascular disease led to the therapeutic development of PCSK9 inhibitors at an unprecedented pace. However, there remain many gaps in our understanding of PCSK9 regulation and biology, including its posttranscriptional control by microRNAs. Using a high-throughput region(3′-UTR) of human microRNA library screen, we identified microRNAs targeting the 3′ untranslated region of human PCSK9. The top 35 hits were confirmed by large-format PCSK9 3′-UTR luciferase assays, and 10 microRNAs were then selected for further validation in hepatic cells, including effects on PCSK9 secretion and LDLR cell surface expression. These studies identified seven novel microRNAs that reduce PCSK9 expression, including miR-221-5p, miR-342-5p, miR-363-5p, miR-609, miR-765, and miR-3165. Interestingly, several of these microRNAs were also found to target other genes involved in LDLR regulation and potently upregulate LDLR cell surface expression in hepatic cells. Together, these data enhance our understanding of post-transcriptional regulators of PCSK9 and their potential for therapeutic manipulation of hepatic LDLR expression.

## Introduction

Cholesterol homeostasis is essential for human health, and its dysregulation results in cardiometabolic diseases, including atherosclerosis. Cells must maintain membrane cholesterol within a narrow concentration to ensure proper membrane function, and this requires an intricate balance of cholesterol synthesis and uptake of cholesterol from plasma lipoproteins (1). Hepatocytes play a major role in the regulation of systemic cholesterol homeostasis through the assembly and secretion of plasma lipoproteins, as well as their eventual clearance through the low-density lipoprotein (LDL) receptor. This highly synchronized process is achieved through a complex network of regulatory and counter-regulatory mechanisms that function at both the transcriptional and post-transcriptional levels. Imbalances in hepatic cholesterol synthesis and uptake can result in elevated levels of LDL cholesterol (LDL-C), a strong, independent risk factor for atherosclerotic cardiovascular disease (ASCVD). Thus, the regulatory networks that maintain cholesterol homeostasis have been the subject of intense research efforts for over 30 years, and discoveries in this area have had major impacts on the management of cardiovascular disease.

Statin drugs have revolutionized the routine management of patients with high LDL-C, significantly lowering the associated cardiovascular morbidity and mortality (2, 3). Statins act to shut off cellular cholesterol biosynthesis by inhibiting 3-HMG-CoA reductase (HMGCR) (4), the rate-limiting enzyme in cholesterol homeostasis. This reduces hepatic cholesterol synthesis, leading to feedback activation of sterol regulatory element (SRE)-binding protein (SREBP), a transcription factor that drives LDL receptor (LDLR) expression (5). The upregulation of hepatic LDLR expression results in increased clearance of circulating LDL, effectively lowering its plasma concentrations and its subsequent negative effects in blood vessels (6). A second major breakthrough came with the identification of proprotein convertase subtilisin/kexin type 9 (PCSK9), a circulating protein that binds to the LDLR and internalizes it for lysosomal degradation (7). The discovery of PCSK9 emerged from seminal studies by Abifadel et al. of a French family with familial hypercholesterolemia, with no known mutations in genes coding for LDLR or its trafficking (8). These patients were found to have gain-of-function mutations in PCSK9, resulting in low levels of hepatic LDLR and LDL clearance. Subsequent studies identified loss-of-function mutations in PCSK9 associated with lifelong low levels of LDL-C and marked reduction in the risk of ASCVD (9–11). This fueled the rapid clinical development of PCSK9 inhibitors, with Food and Drug Administration (FDA) approval of human monoclonal antibodies that bind PCSK9 and reduce LDL-C by 60% (12), occurring in record time less than a decade after the discovery of PCSK9. Despite this success, there is still much to be learned about PCSK9's regulation and function, which may inform not only additional facets of PCSK9 biology but also alternative mechanisms of PCSK9 antagonism.

An area of PCSK9 regulation that remains underexplored is its posttranscriptional inhibition by microRNAs (miRNAs). MiRNAs are short (20–25 base pairs) non-coding RNA sequences that are transcribed from the genome and are capable of binding to complementary sequences in the 3′ untranslated region (3′-UTR) of mRNA transcripts (13). MiRNA targeting of mRNAs results in posttranscriptional inhibition of their translation into protein and/or their degradation (14–16). Previous studies have shown the potential for a handful of miRNAs to directly inhibit the expression and function of PCSK9 in human or murine hepatocytes, including miR-224, miR-191, miR-222, miR-483, and miR-520d (17–20). These studies have uncovered new links for PCSK9 with disease, as well as how miRNAs can integrate the regulation of multiple genes involved in cholesterol metabolism. For example, miR-224 was identified as a potent inhibitor of PCSK9 expression in hepatocytes (18) and subsequently found to regulate PCSK9 in human neuroendocrine tumors (20), where its overexpression restricted tumor cell proliferation and invasion (20). Further studies showed that miR-224 represses not only PCSK9 but also other genes impacting the expression of the LDLR, including HMGCR and a second LDLR chaperone protein called IDOL (19). Notably, hepatic overexpression of miR-224 in mice using nanoparticle mediated delivery of miR-224 mimics decreased plasma LDL-C levels by 15% (19). Similarly, miR-483 was shown to repress expression of PCSK9 in hepatocytes, leading to upregulation of the LDLR and LDL uptake, and adeno-associated virus-mediated delivery of miR-483 in mice overexpressing PCSK9 decreased total plasma cholesterol levels by 20% (17). These studies highlight the feasibility of harnessing miRNA-driven regulation of PCSK9 expression to regulate plasma levels of LDL-C *in vivo*.

To identify novel regulators of PCSK9 expression in humans, we performed a high-throughput screen of human miRNAs targeting the 3′-UTR of *PCSK9* fused to a luciferase gene. Candidate miRNAs that repressed *PCSK9* 3′-UTR luciferase activity were selected using a strictly standardized mean difference (SSMD) analysis of the relative changes in sample luciferase activities and cross-referenced with miRNA target algorithms such as TargetScan and miRanda. The resulting miRNA hits underwent secondary validation in miRNA mimic transfection assays to examine their effect on *PCSK9* mRNA and protein levels in hepatic cell lines, and the top 10 miRNAs were selected for validation assays, including regulation of LDLR protein expression and cell surface localization. Notably, several candidate miRNAs identified to target *PCSK9* were also found to target genes involved in other pathways that regulate LDLR cell surface expression (i.e., *HMGCR* and *IDOL*), suggesting that targeting such miRNAs may have a higher therapeutic impact.

## Materials and Methods

### Cell Culture

HepG2 and HEK-293T cells were obtained from the American Type Culture Collection, authenticated with standard American Type Culture Collection methods (morphology check under microscope and growth curve analysis) and regularly tested for mycoplasma contamination. Cells were maintained in Dulbecco's modified Eagle medium (DMEM; Corning, New York, NY, USA) containing 10% fetal bovine serum (Life Technologies, Carlsbad, CA, USA) and 1% penicillin–streptomycin (Life Technologies). HepG2-LDLR-GFP cells were grown as previously described (21). All cells were cultured in a humidified incubator at 37°C and 5% CO_2_.

### High-Throughput Human MicroRNA Library Screens

#### Primary Screen

A high-throughput human miRNA screen was conducted with the NYU RNAi Core Facility (NYU Grossman School of Medicine). HEK-293T cells were reverse transfected in triplicate with a library of 1,719 miRNA mimics (Life Technologies *mir*Vana Mimic Library, miRbase release 17.0) in Corning 384-well, flat, white-bottom polystyrene TC-treated microplates. Briefly, 5,000 cells/well, 1.5 pmol of miRNA mimic, and 10 ng of total DNA plasmids composed of a 2:1 ratio of *PCSK9* 3′-UTR firefly luciferase reporter plasmid (SwitchGear Genomics, Carlsbad, CA, USA) to renilla luciferase control plasmid using Lipofectamine 2000 (Invitrogen, Carlsbad, CA, USA) were dispensed into the 384-well plates using a WellMate/BioTek Dispenser (Thermo Fisher Scientific, Waltham, MA, USA) outfitted with small-bore tubing. The plates were centrifuged at 1,000 × g for 1 min to settle the contents and then incubated in a humidified incubator at 37°C and 5% CO_2_ for 48 h. To measure *PCSK9* 3′-UTR-luciferase activity, media were aspirated, and the wells were washed with 1 × phosphate-buffered saline (PBS) using an EL406 Microplate Washer (BioTek). Luciferase activity was measured using the Dual-Glo Luciferase Assay® (Promega, Madison, WI, USA) according to the manufacturer's instructions and read on a spectrophotometer (EnVision; PerkinElmer, Waltham, MA, USA). Positive (miR-224) and negative control miRNAs were included on each screen plate, and the miRNA library was assayed in quadruplicate. *PCSK9* 3′-UTR firefly luciferase values were normalized to those of control renilla luciferase values for each replicate. Normalized firefly luciferase activities were ranked using the SSMDs of log(N^exp^/N^plate.avg^), Z-score (N^exp^-N^plate.avg^/SD N^plate.avg^), and percent of negative control (POC), relative to plate averages as previously described (22). MiRNA candidates with a composite SSMD score of < -1 were identified as hits.

#### Secondary Screen

Target prediction algorithms were used to validate putative binding to the human *PCSK9* 3′-UTR, and the top 35 screen hits were selected for a secondary screen, consisting of a similar but larger-format luciferase assay. HEK-293T cells were plated in antibiotic-free media in 24-well plates and co-transfected with 0.2 μg of 3′-UTR luciferase *PCSK9* reporter vector and miRNA mimic or negative control mimic (Dharmacon; Horizon Discovery, Cambridge, UK) utilizing Lipofectamine 2000 (Invitrogen), as previously described. Luciferase activity was measured using the Dual-Glo Luciferase Assay® (Promega). Firefly luciferase activity was normalized to renilla luciferase activity and reported as fold change in activity compared with control mimic. Experiments were performed in triplicate wells of a 24-well plate and repeated three times.

### Transfection of Hepatic Cells With MicroRNA Mimics

Human hepatic cells (HepG2) were plated at a density of 10^6^ cells per well on 6-well cell culture plates and were transfected with control or miRNA mimics (80 nM, Dharmacon) using Lipofectamine® RNAi MAX (Invitrogen). After 48 h, cells were harvested and lysed with either TRIzol Reagent (Invitrogen) for RNA expression analysis or radioimmunoprecipitation assay (RIPA) buffer (Abcam, Cambridge, UK) for protein expression analysis.

### RNA Isolation and Quantitative RT-PCR

Total RNA was isolated with TRIzol Reagent and Direct-zol RNA MiniPrep Columns (Zymo Research, Irvine, CA, USA). RNA was reverse transcribed using iScript™ cDNA Synthesis Kit (Bio-Rad Laboratories, Hercules, CA, USA), according to the manufacturer's protocol. Quantitative RT-PCR analysis was conducted using iQ Sybr Green Supermix (Bio-Rad) in a Mastercycler PCR Machine (Eppendorf, Hamburg, Germany) using the following primers: PCSK9 forward 5′-AGGGGAGGACATCATTGGTG-3′ and reverse 5′-CAGGTTGGGGGTCAGTACC-3′; HMGCR forward 5′-GTCATTCCAGCCAAGGTTGT-3′ and reverse 5′-GGGACCACTTGCTTCCATTA-3′; IDOL forward 5′-CGAGGACTGCCTCAACCA-3′ and reverse 5′-TGCAGTCCAAAATAGTCAACTTCT-3′; and GAPDH forward 5′GAAGGTGAAGGTCGGAGTC-3′ and reverse 5′-GAAGATGGTGATGGGATTTC-3′. Fold change in mRNA expression was calculated with the comparative cycle method (2^−Δ*ΔCt*^) and normalized to the housekeeping gene *GAPDH*.

### Western Blotting Analysis and ELISA

PCSK9 (CY-P1037; MBL International Corporation, Woburn, MA, USA) and LDLR (1007665; Cayman Chemical, Ann Arbor, MI, USA), and GAPDH (G9545, Sigma-Aldrich, St. Louis, MO, USA) antibodies were used for immunoblotting of lysates prepared from HepG2 cells transfected with miRNA mimics. Protein bands were visualized using the Odyssey Infrared Imaging System (LI-COR Biosciences, Lincoln, NE, USA). Densitometry analysis of the gels was carried out using ImageJ software from the National Institutes of Health (NIH) (http://rsbweb.nih.gov/ij/). To measure secreted PCSK9 levels, HepG2 cells were transfected with miRNA mimics and incubated in DMEM containing 5% lipoprotein-deficient serum. After 24 h, the culture medium was collected and centrifuged to remove cellular debris. Cell supernatants were assayed by human PCSK9 Quantikine Enzyme-Linked Immunosorbent Assay (R&D Systems, Minneapolis, MN, USA).

### Immunofluorescence Microscopy

HepG2 cells constitutively expressing LDLR-GFP were obtained from Peter Tontonoz (University of California, Los Angeles). Cells were resuspended in 10% lipoprotein-deficient serum (LPDS), plated in chamber slides (LabTekII; Thermo Fisher Scientific) and transfected with miRNA mimics (80 nM, Dharmacon) using Lipofectamine® RNAi MAX (Invitrogen). After 48 h, the cells were washed and fixed with 4% paraformaldehyde and stained with DAPI nuclear stain (D-9542, Sigma-Aldrich) for 10 min. After washing, cells were mounted with coverslips using mounting medium for fluorescence (H-1000; Vector Laboratories Inc., Burlingame, CA, USA). Fluorescent images were collected with an LSM 510 confocal laser-scanning microscope (Carl Zeiss, Oberkochen, Germany) with 63 × /1.4 oil objective. The frame size was 1,024 × 1,024. The manufacturer's software was used for data acquisition and ImageJ for fluorescence profiles. The weighted colocalization coefficients were calculated using AIM (Carl Zeiss). The cells were visualized with an Axiovert 25 (Carl Zeiss) with a 10 × /0.25 or 32 × /0.40 objective.

### Statistical Analysis

Screen data were analyzed using SSMDs of percent of control, Z-score, and log(N^well^/N^plate.avg^), where N represents firefly luciferase reporter activity divided by renilla luciferase activity in the corresponding well and N^plate.avg^ represents the average firefly reporter activity normalized to renilla luciferase control for the whole plate. The SSMD values were averaged and ranked across the entire screen. Validation data are presented as mean ± standard error of the mean (s.e.m) (*n* is noted in the figure legends). Statistical significance of differences was evaluated with an unpaired two-sided Student's *t*-test or one-way ANOVA (as indicated in the figure legends). Significance was accepted at the level of *p* < 0.05. Data analysis was performed using GraphPad Prism Software (GraphPad, La Jolla, CA, USA).

## Results

### A High-Throughput Screen Identifies MicroRNA Modulators of Human PCSK9

**Figure 1:**
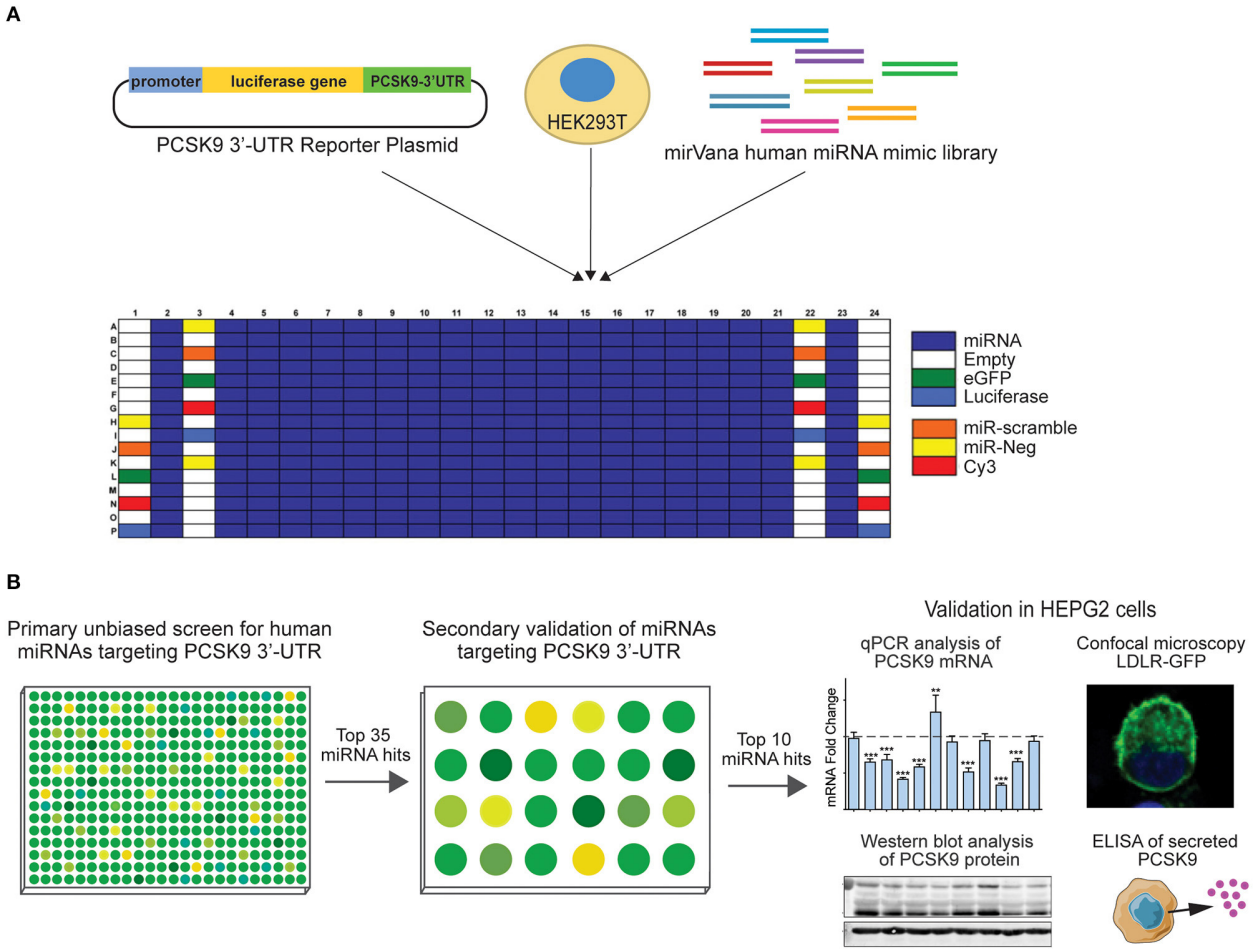
Experimental design for high-throughput human miRNA library screen to identify novel miRNAs targeting *PCSK9*. **(A)** Primary unbiased screen of human miRNAs targeting the 3′-UTR of human *PCSK9*. HEK-293T cells underwent reverse transfection with a luciferase reporter plasmid harboring the 3′-UTR of *PCSK9*, and a human miRvana miRNA mimic library in a 384-well array format. **(B)** Secondary screen of the top 35 miRNA hits using the same *PCSK9* 3′-UTR-luciferase assay in a 24-well format and further validation of the top 10 candidates for effects on PCSK9 mRNA and protein expression, and low-density lipoprotein receptor (LDLR) expression.

**Figure 2:**
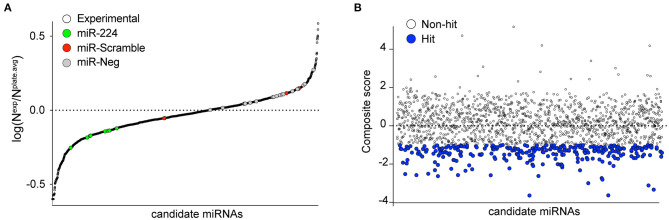
Primary screening of human miRNAs targeting the 3′-UTR of *PCSK9*. **(A)** MiRNAs were tested for their ability to repress a luciferase reporter gene fused to the 3′-UTR of human *PCSK9*. Data represent average log normalized scores [log(N^exp^/N^plate.avg^)] of luciferase activity of three replicates. N^exp^ represents firefly reporter activity normalized to renilla luciferase control per well. N^plate.avg^ represents the average firefly reporter activity normalized to renilla luciferase control for the whole plate. Negative (miR-scramble and miR-negative) and positive (miR-224) controls are indicated by the colored dots. **(B)** Ranking of miRNA hits by composite score of the strictly standardized mean differences (SSMDs) of log(N^exp^/N^plate.avg^), Z-score, and percentage of negative control (POC). MiRNAs with composite SSMD scores of < -1 were identified as positive hits (blue) in the screen.

**Figure 3:**
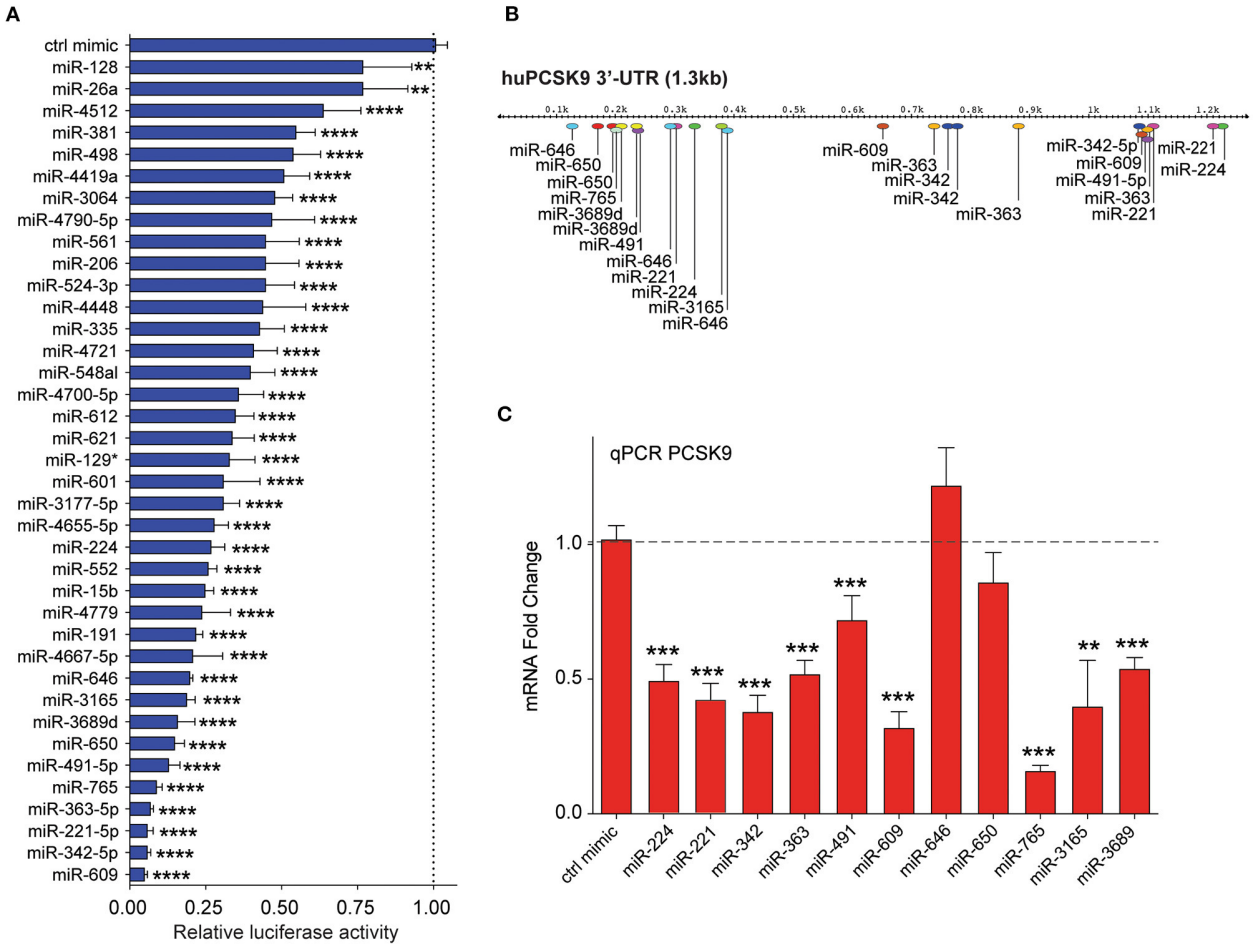
Secondary screening of top miRNA candidates targeting human *PCSK9*. **(A)** Relative luciferase activity of *PCSK9* 3′-UTR luciferase reporter construct in HEK-293T cells transfected with miRNA mimics for the top 35 hits identified from the primary screen. MiR-224 was included as a positive control. Data are presented as percent of control mimic ± standard deviation of three independent experiments. **(B)** Alignment of miRNA binding sites for the top 10 candidate miRNAs on the human *PCSK9* 3′-UTR. **(C)** qRT-PCR analysis of *PCKS9* expression in HepG2 cells transfected with the top 10 candidate miRNAs or control mimic. Data are the mean ± standard deviation from three independent experiments. *p*-Values were calculated by one-way ANOVA and corrected for multiple comparison by Dunnet's *post-hoc* test **(A)** or a two-tailed Student's *t*-test **(C)**. *p < 0.05, **p < 0.01, ***p < 0.001, ****p < 0.0001.

To identify putative miRNA candidates controlling the expression of human *PCSK9*, we conducted an unbiased screen for human miRNAs, which could modulate the activity of a firefly luciferase reporter vector harboring the 3′-UTR of *PCSK9*. The experimental design of the high-throughput 384-well based primary screen is shown in Figure 1A. HEK-293T cells were co-transfected with the human *PCSK9* 3′-UTR-luciferase reporter and a renilla luciferase control plasmid, along with a library of human miRNA mimics. Each plate included negative (miR-scramble, miR-Neg, eGFP, Cy3, and miR-21) and positive (miR-224) control wells. Forty-eight hours after transfection, firefly and renilla luciferase activities were measured in each well, and normalized firefly luciferase activities were ranked using SSMDs of percent of control and log values, relative to plate averages (Figure 2A). Approximately 100 microRNAs with composite scores of < -1 were identified as positive hits in the assay, indicating strong targeting of the 3′-UTR of *PCSK9* by these miRNAs (blue dots, Figure 2B, Supplementary Table 1). Corroborating the screen's robustness, we identified previously characterized miRNA modulators of *PCSK9*, including miR-191 and miR-224 (18, 19), as potent inhibitors of *PCSK9* 3′-UTR-luciferase activity (Figure 3A).

By integrating the miRNA screen results with target prediction algorithms to confirm *PCSK9* targeting (Supplementary Figure 1), we identified 35 miRNAs for secondary screening using larger-format 24-well plate luciferase activity assays. As in the primary assay, HEK-293T cells were co-transfected with the human *PCSK9* 3′-UTR luciferase reporter, control renilla luciferase plasmid, and miRNA mimics or control; and luciferase activity was measured 48 h later. Of the 35 miRNAs identified in the primary screen, all were confirmed to reduce *PCSK9* 3′-UTR luciferase activity by 25% or greater, with 30/35 miRNAs reducing luciferase activity by more than 50% (Figure 3A). The top 10 miRNAs, which were found to repress *PCSK9* 3′-UTR luciferase activity by more than 80%, and the positive control miRNA miR-224 (19) were selected for further functional screening in hepatic cells. The full experimental design is summarized in Figure 1B.

### Endogenous RNA and Protein PCSK9 Are Altered by MicroRNAs Unveiled in Initial Screens

**Figure 4:**
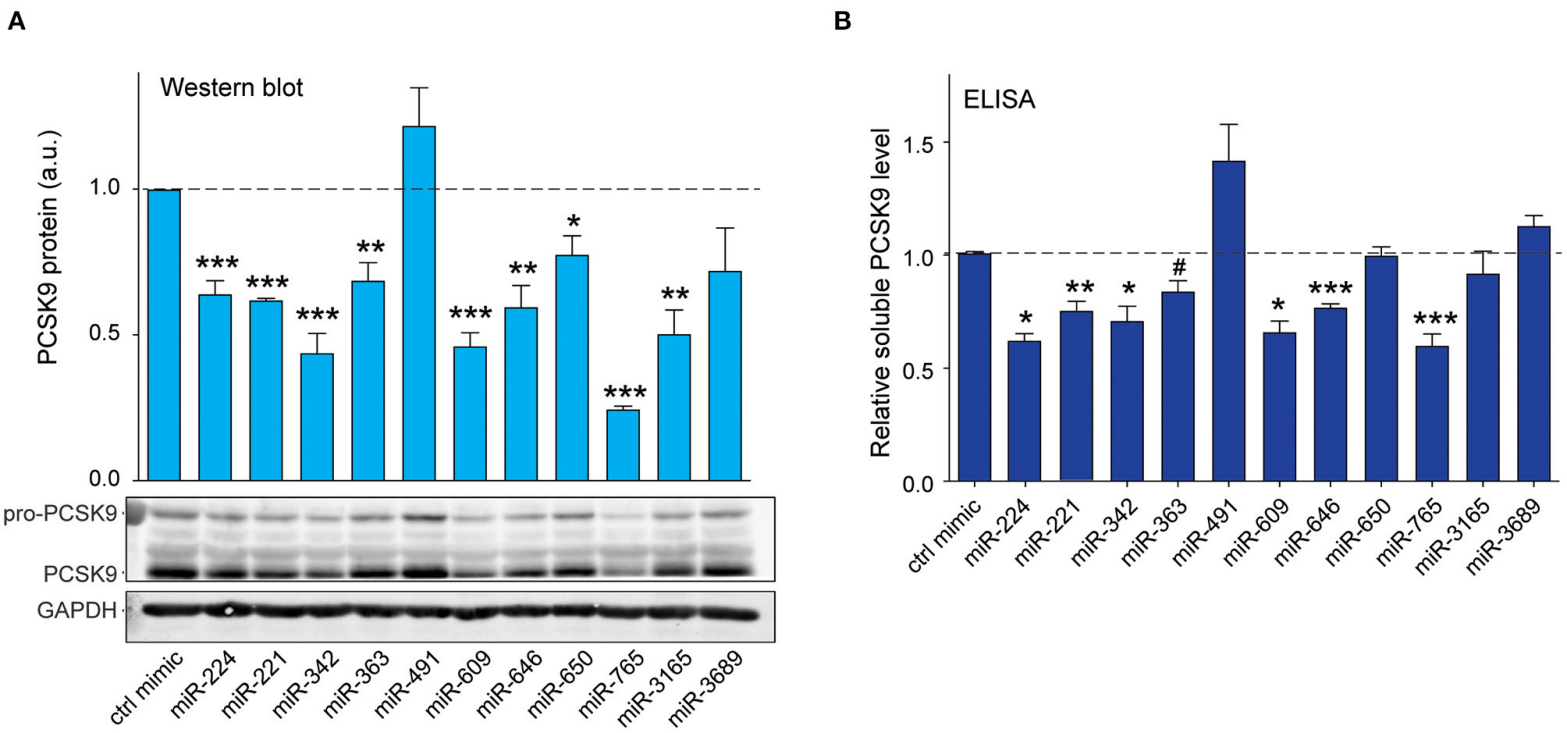
Validation of top 10 candidate miRNAs targeting human PCSK9. **(A)** Western blotting analysis of PCSK9 and GAPDH protein in HepG2 cells transfected with miRNA mimics or a control mimic. Relative quantification of three independent experiments is shown above the blots. **(B)** ELISA quantification of secreted PCSK9 in supernatants derived from HepG2 cells transfected with miRNA mimics or a control mimic. **(A,B)** Data are the mean ± standard error of the mean from three independent experiments. *p*-Values were calculated using two-tailed Student's *t*-test. ^#^*p* < 0.1, *p < 0.05, **p < 0.01, ***p < 0.001.

Mapping of the binding sites of top 10 *PCSK9*-targeting miRNAs to its 3′-UTR showed that eight of these had two or more predicted binding sites within the 3′-UTR (miR-221-5p, miR-342-5p, miR-363-5p, miR-491, miR-609, miR-646, miR-650, and miR-3689), as did the positive control miRNA miR-224. This analysis identified hot spots for predicted miRNA binding clustering near both the 5′ and 3′ ends of the UTR (Figure 3B). To test the functionality of these binding sites in regulating endogenous PCSK9 expression, we transfected HepG2 cells with miR-mimics for each of the 11 miRNA candidates or a control miRNA, and we measured *PCSK9* mRNA by qRT-PCR and protein levels by Western blotting. Mimics for miR-224, miR-221, miR-342, miR-363, miR-491, miR-609, miR-765, miR-3165, and miR-3689 significantly decreased *PCSK9* mRNA levels, as compared with control mimic (Figure 3C). Despite the presence of three putative binding sites for miR-646 and two putative binding sites for miR-650 in the *PCSK9* 3′-UTR, overexpression of these miRNAs did not reduce *PCSK9* mRNA levels in HepG2 cells. Analysis of total cellular PCSK9 protein by Western blotting showed that miR-224, miR-221, miR-342, miR-363, miR-609, miR-646, miR-650, miR-765, and miR-3165 significantly reduced PCSK9 protein levels as compared with control mimic (Figure 4A). Interestingly, while mimics for miR-646 and miR-650 showed no effect on the mRNA levels of *PCSK9*, they decreased PCSK9 protein levels by 40 and 25%, respectively (Figure 4A), suggesting that binding of miR-646 and miR-650 to the 3′-UTR of *PCSK9* may lead to repression of mRNA translation, but not to degradation of the *PCSK9* transcript.

As PCSK9 is secreted by hepatocytes and modulates cell surface expression of LDLR by binding to the extracellular portion of the receptor, we next measured the effects of the top 10 miRNA candidates on soluble PCSK9 elaborated by HepG2 cells. We treated HepG2 cells with control or miRNA mimics and collected cell culture supernatants 24 h later for analysis by ELISA. Significant reductions in soluble PCSK9 were observed in HepG2 cells expressing miR-224, miR-221, miR-342, miR-363, miR-609, miR-646, and miR-765 compared with control mimics (Figure 4B). Consistent with our Western blotting results showing that miR-491 and miR-3689 did not significantly reduce total cellular PCSK9 protein levels in HepG2 (Figure 4A), these miRNAs also failed to reduce PCSK9 secretion by HepG2 cells (Figure 4B). Collectively, these results identify miR-221, miR-342, miR-363, miR-609, miR-646, miR-765, and miR-3165 as novel regulators of PCSK9.

### PCSK9-Targeting MicroRNAs Regulate Low-Density Lipoprotein Receptor Protein Expression and Cellular Distribution

**Figure 5:**
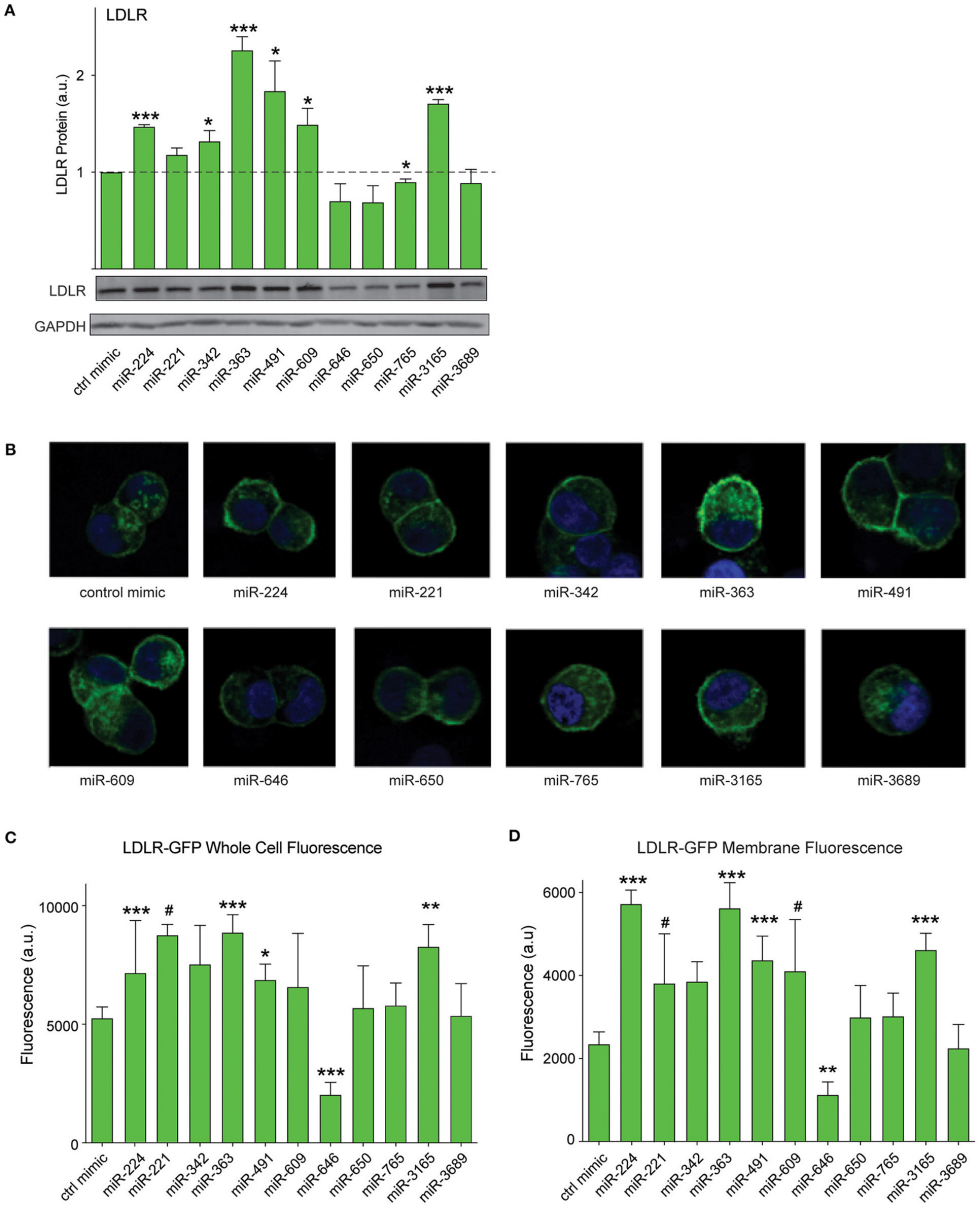
PCSK9-targeting miRNAs regulate hepatic low-density lipoprotein receptor (LDLR) expression. **(A)** Western blotting analysis of LDLR and GAPDH protein in HepG2 cells transfected with miRNA mimics or a control mimic. Relative quantification of three independent experiments (mean ± standard error of the mean) is shown above the blots. **(B)** Representative fluorescent images of HepG2 cells stably expressing LDLR-GFP (green) transfected with miRNAs mimics or a control miR mimic. Counterstaining with DAPI to visualize nucleus (blue). **(C,D)** Quantification of **(C)** whole cell or **(D)** cell surface LDLR-GFP fluorescence intensity in HepG2 cells transfected with miRNA mimics or a control miR mimic. Data are representative of three independent experiments. *p*-Values were calculated using two-tailed Student's *t*-test. ^#^*p* < 0.1, *p < 0.05, **p < 0.01, ***p < 0.001.

As PCSK9 can bind to the LDLR and promote its degradation, we next tested whether the PCSK9-targeting miRNAs could alter total LDLR expression in HepG2 cells. We transfected HepG2 cells with the top 10 miRNAs identified in our screen or positive (miR-224) and negative (control mimic) control miRNAs, and we measured cellular LDLR protein by Western blotting analysis. Transfection of mimics for miR-224, miR-342, miR-363, miR-491, miR-609, and miR-3165 increased LDLR protein levels compared with control mimics (Figure 5A). Next, to understand whether these *PCSK9*-targeting miRNAs affect LDLR cell surface expression, we used HepG2 cells stably expressing a GFP-tagged LDLR (HepG2-LDLR-GFP). The LDLR-GFP transgene does not contain the native LDLR 3′-UTR and thus cannot be directly targeted by the miRNA mimics. Transfection of HepG2-LDLR-GFP cells with miR-224, miR-363, miR-491, and miR-3165 increased both whole cell fluorescence and membrane-specific expression of LDLR-GFP when compared with control mimics (Figures 5B–D). Similar trends in LDLR-GFP upregulation were observed with miR-221 and miR-609, but the increase did not reach statistical significance (Figures 5B–D).

**Figure 6:**
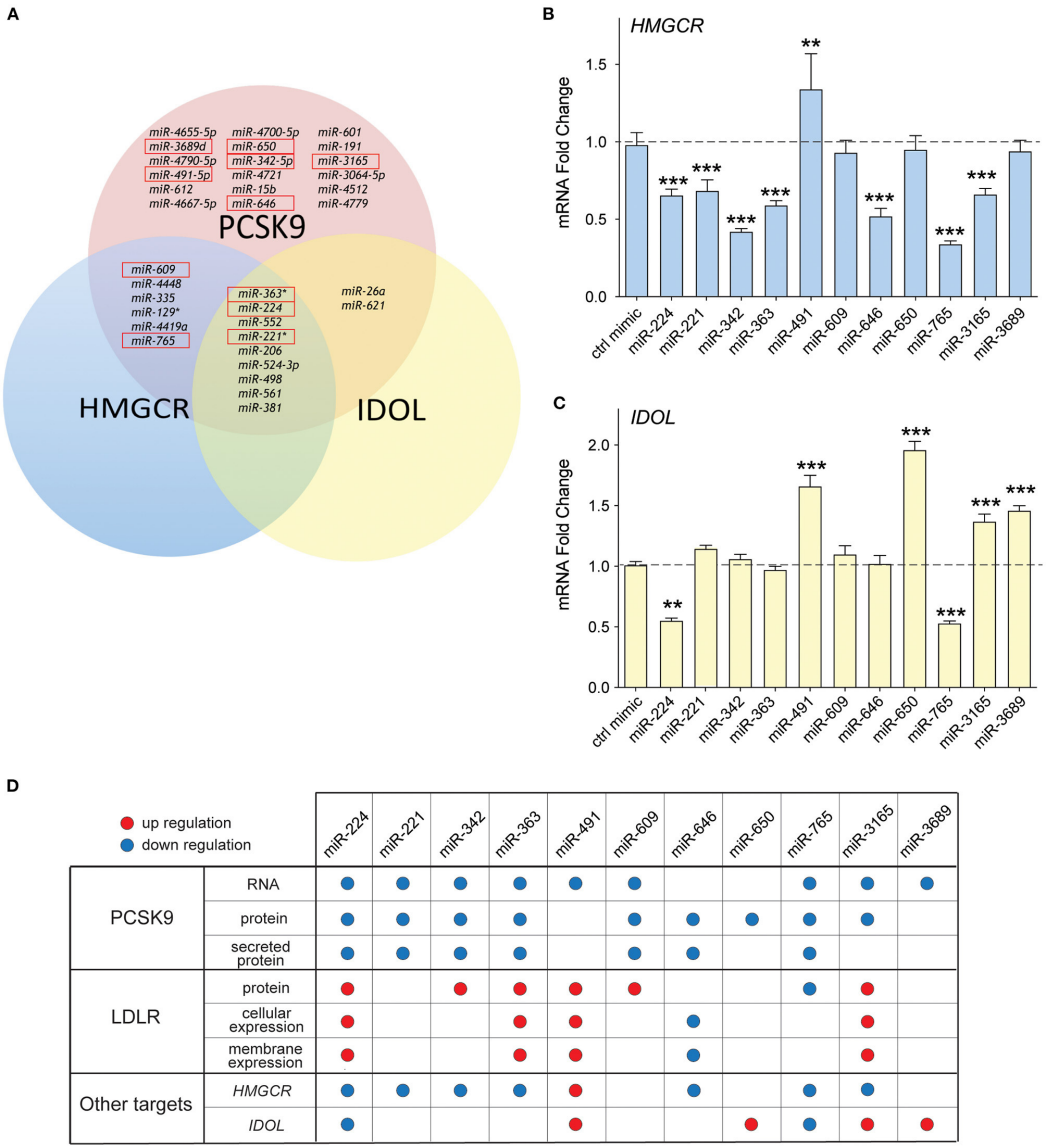
Common targeting of genes involved in low-density lipoprotein (LDL) receptor regulation by candidate miRNAs. **(A)** Venn diagram of the top 35 miRNAs identified to repress *PCSK9* showing overlapping targeting of *HMGCR* and *IDOL*. The top 10 miRNAs validated for PCSK9 targeting are shown in the red boxes. **(B,C)** qRT-PCR analysis of **(B)** *HMGCR* and **(C)** *IDOL* mRNA in HepG2 cells transfected with the top 10 candidate miRNA mimics or a control miR mimic. Data are the mean ± standard error of the mean from three independent experiments. *p*-Values were calculated using two-tailed Student's *t*-test. **p < 0.01, ***p < 0.001. **(D)** Table summarizing the effects for the top 10 miRNAs targeting PCSK9. Red and blue dots represent significant upregulation or downregulation, respectively.

Previous studies from our group showed that in addition to PCSK9, miR-224 targets two additional genes known to modulate LDLR abundance (19): *IDOL*, the chaperone protein that mediates proteasomal degradation of the LDLR (23), and *HMGCR*, the rate-limiting enzyme in cholesterol biosynthesis (4). Interestingly, bioinformatics analysis of the top miRNA hits from our screen showed that 9/35 miRNAs were predicted to target *PCSK9, IDOL*, and *HMGCR*, while 6/35 were predicted to target *PCSK9* and *HMGCR* (Figure 6A). Included among those miRNAs predicted to repress multiple targets involved in LDLR regulation were four from our top 10 list: miR-221, miR-363, miR-609, and miR-765. To investigate whether our top 10 miRNA candidates also regulate *HMGCR* or *IDOL* expression, we transfected HepG2 cells with miRNA mimics and measured mRNA levels of these genes by qPCR. We observed significant downregulation of *HMGCR* mRNA by miRNA-224, miR-221, miR-342, miR-363, miR-646, miR-765, and miR-3165 when compared with control mimics (Figure 6B). By contrast, only two miRNAs, miR-224 and miR-765, reduced *IDOL* mRNA levels in HepG2 cells (Figure 6C). Collectively, these data suggest that miR-221, miR-342, miR-363, and miR-3165 may alter hepatic expression of the LDLR by dual targeting of *PCSK9* and *HMGCR* (Figure 6D).

## Discussion

The discovery that loss-of-function mutations in PCSK9 are associated with lifelong low cholesterol levels and protection from ASCVD sparked intense efforts to develop inhibitors of this circulating protein (7). The first approved PCSK9 inhibitors, human monoclonal antibodies that bind extracellular PCSK9, show remarkable efficacy in reducing LDL-C either as monotherapy (50% reduction) or in combination with a statin (70% reduction) (24). Although highly effective, these monoclonal antibodies require injections every 2–4 weeks, and additional approaches for PCSK9 inhibition are being actively pursued (25). MiRNAs have emerged as exciting new therapeutic targets for manipulation of metabolic pathways, including cholesterol homeostasis (26). In this study, we used a high-throughput screening strategy to identify miRNA regulators of human PCSK9. Using a screening pipeline of 3′-UTR-reporter assays, followed by expression and validation assays, we identify seven novel miRNAs targeting *PCSK9*: hsa-miR-221-5p, hsa-miR-342-5p, hsa-miR-363-5p, hsa-miR-609, hsa-miR-646, hsa-miR-765, and hsa-miR-3165. Furthermore, using measurements of whole cell LDLR protein and a fluorescent LDLR-GFP cell line, we show that miR-221, miR-342, miR-363, and miR-3165 can heighten expression of the LDLR in hepatic cells, validating the effects of these miRNAs in short-circuiting the effect of PCSK9 on endosomal LDLR recycling. Notably, miR-221, miR-342, miR-363, and miR-3165 were also identified to repress *HMGCR*, which would be expected to trigger SREBP-mediated upregulation of LDLR expression, thereby reinforcing the effects of PCSK9 inhibition. Such miRNAs, which simultaneously target multiple genes involved in regulating LDLR cell surface expression, may offer greater therapeutic potential for regulating LDL-C.

Although the development of PCSK9 inhibitors has moved at an unprecedented pace, there is still much to learn about PCSK9 regulation and biology. At the transcriptional level, PCSK9 has been shown to be regulated by an SRE motif within its promoter (27, 28). Depletion of intracellular cholesterol levels causes translocation of the SREBP transcription factor to the nucleus and transcriptional upregulation of genes involved in cholesterol synthesis and uptake, such as LDLR. Paradoxically, SREBP also upregulates PCSK9, which blunts cholesterol uptake through the LDLR by causing its internalization (29, 30). This is particularly relevant in the setting of statin treatment, where SREBP-mediated upregulation of PCSK9 attenuates LDL-C lowering (29, 30) and why combination therapies of PCSK9 inhibitors with statins are attractive for optimal lipid lowering. In addition to SREBP, the hepatic nuclear factor 1alpha (HNF1α) and peroxisome proliferator-activated receptor gamma (PPARγ) have been shown to contribute to transcriptional regulation of PCSK9 (31, 32). By contrast, relatively little is known about the posttranscriptional mechanisms regulating PCSK9. Previous studies have identified roles for various miRNAs, including miR-224, miR-191, miR-222, miR-483, and miR-520d, in inhibiting the expression and function of PCSK9 in human or murine hepatocytes (17–20), but to our knowledge, this is the first study to perform a systematic screen of miRNAs targeting the human *PCSK9* 3′-UTR. Of the top 35 miRNAs selected for secondary screening, 10 were selected for further validation. Although all 10 miRNAs showed potent repressive effects on the activity of a *PCSK9* 3′-UTR-luciferase reporter (>75%), only seven of these were confirmed to reduce PCSK9 mRNA and/or protein in hepatic HepG2 cells: miR-221, miR-342, miR-363, miR-609, miR-646, miR-765, and miR-3165. Notably, none of these miRNAs have previously been shown to target PCSK9, highlighting the utility of such a screen in identifying novel posttranscriptional regulators of genes of interest.

Therapeutically, the most interesting miRNAs would be ones that not only repress PCSK9 secretion but also show efficacy in upregulating hepatic LDLR surface expression. In that category, miR-221, miR-342, miR-363, and miR-3165 were the most promising candidates. Interestingly, miR-221, miR-342, and miR-363 are each predicted to have three miRNA binding sites within the *PCSK9* 3′-UTR, including sites clustered near the 3′ end of the UTR. To date, no studies have reported links between miR-221 or miR-363 and cholesterol metabolism. However, a recent report showed that miR-342 is upregulated following viral infection and induces a coordinate reduction in the abundance of many genes in the sterol biosynthesis pathway (33), including its master regulator *SREBF2*, as well as *HMGCR* as we showed herein. This type of pathway modulation by miRNAs provides a potent mechanism for synchronized inhibition of gene pathways (34) and also illustrates the complexity of miRNA-mediated posttranscriptional regulatory networks. Although we focused on miRNA repression of human PCSK9 in our study, targeting of PCSK9 by mmu-miR-221-5p, mmu-miR-363-5p, and mmu-miR-342-5p is conserved in mice, which would allow future *in vivo* studies of these miRNAs in mice. Such studies would provide information on whether miR-221, miR-363, and miR-342 targeting of hepatic PCSK9 expression is conserved in mice and whether these miRNAs alter plasma levels of LDL-C.

RNA-based therapeutics against PCSK9 hold promise as alternatives to monoclonal antibody therapy, which requires frequent dosing. Although no miRNA-based therapies are currently in the pipeline, a synthetic siRNA directed against PCSK9, inclisiran, is in advanced stages of development. Inclisiran engages the endogenous RNA interference (RNAi) pathway by binding to the RNA-induced silencing complex (RISC) and enabling cleavage of the *PCSK9* mRNA (35). One potential advantage of this approach, which would also be shared by miRNA-based therapeutics, is that it inhibits the intracellular production of PCSK9 prior to its secretion. Whereas monoclonal antibodies cause significant accumulation of PCSK9-bound antibodies, siRNA- or miRNA-mediated knockdown of *PCSK9* reduces protein production by hepatocytes, similar to what is observed with PCSK9 loss-of-function mutations. Synthetic RNAs can also be conjugated to triantennary *N*-acetylgalactosamine carbohydrates to enhance hepatic uptake via asialoglycoprotein receptors and show an extended duration of action. Indeed, in Phase II clinical trials, two doses of inclisiran resulted in profound suppression of PCSK9 and LDL-C for at least 6 months (24). The success of this siRNA-based therapy reflects the remarkable improvements in the safety and efficacy of RNA-targeted treatment approaches over the last decade and suggests that miRNA-based therapies might not be far behind.

## Data Availability Statement

The data that support the findings of this study are available from the corresponding author upon request. The datasets presented in this study can be found in online repositories and are available at the Gene Expression Omnibus (GEO) under accession number GSE166680.

## Author Contributions

KM, SO, and CvS designed the study, guided the interpretation of the results, and prepared the manuscript, with input from all authors. SO, AS, and AW performed experiments and data analyses. All authors contributed to the article and approved the submitted version.

## Conflict of Interest

The authors declare that the research was conducted in the absence of any commercial or financial relationships that could be construed as a potential conflict of interest.
