# Supplementary material for: High-Throughput Screening Identifies MicroRNAs Regulating Human PCSK9 and Hepatic Low-Density Lipoprotein Receptor Expression
Source: Front Cardiovasc Med. 2021 Jul 12;8:667298. doi: 10.3389/fcvm.2021.667298 (PMC8310920; doi:10.3389/fcvm.2021.667298)
Supplement: Supplementary file 1 [file Data_Sheet_1.pdf]

Supplementary Material

High throughput screening identifies microRNAs regulating human PCSK9 and hepatic LDL receptor expression

Coen van Solingen[1], Scott R. Oldebeken[1], Alessandro G. Salerno[1,2], Amarylis C. B. A. Wanschel[1,2], Kathryn J. Moore[1,3] *

[1]Leon H. Charney Division of Cardiology, NYU Cardiovascular Research Center, Department of Medicine, New York University School of Medicine. New York, NY, USA 10016.

[2]Current affiliation: University of Miami, Coral Gables, FL USA 33146

[3]Department of Cell Biology, New York University School of Medicine. New York, NY, USA 10016.

*** Correspondence:**
Kathryn Moore
kathryn.moore@nyulangone.org

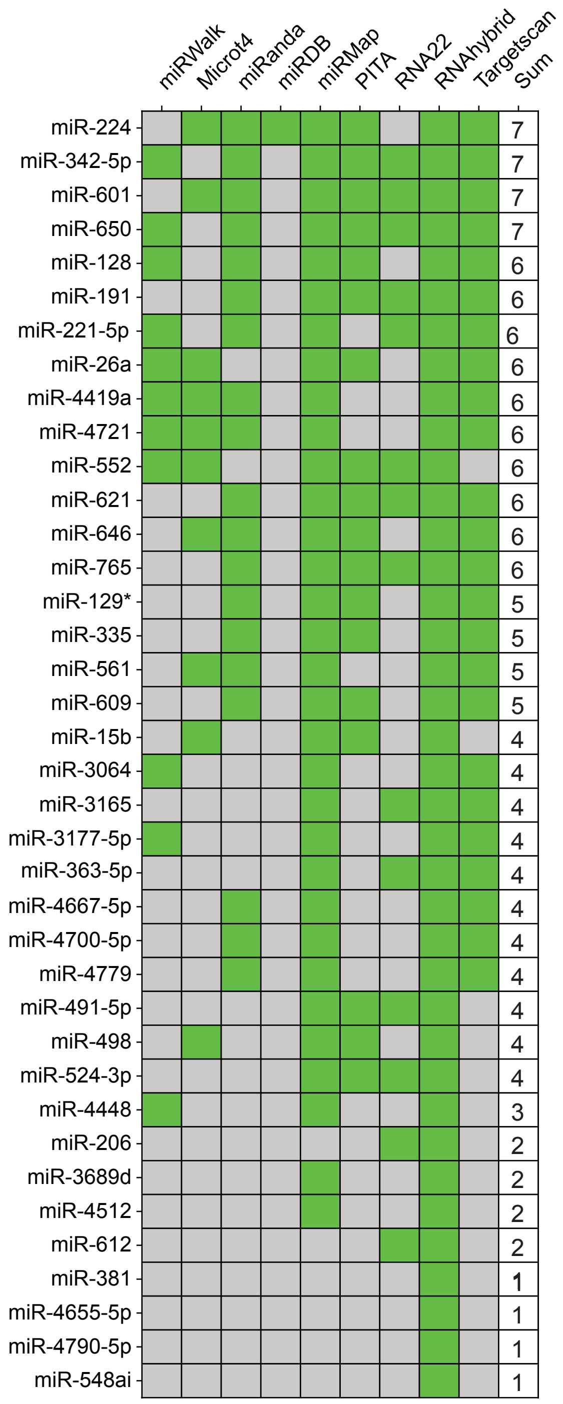

Supplementary Figure I. Prediction of miRNA binding to the human *PCSK9* 3'-UTR for the top 35 miRNAs identified from the primary screen. Green squares indicate a positive prediction of miRNA binding using the indicated miRNA target prediction algorithms. Candidates were ranked based on the sum of positive predictions of miRNA binding sites in the *PCSK9* 3'-UTR.

Supplementary Table I. Top 100 miRNAs identified from primary screen of human miRNA mimics that repress PCSK9 3'-UTR-luciferase reporter activity

Rank	Accession Number(s)	Gene Symbol	Ambion_mirna_id	Ambion_miProd_ID	Mature_Sequence
1	MIMAT0019721	hsa-miR-4655-5p	21783	MC21783	CACCGGGGAUGGCAGAGGGUCG
2	MIMAT0003277	hsa-miR-609	11618	MC11618	AGGGUGUUUCUCUCAUCUCU
3	MIMAT0019049	hsa-miR-4512	22380	MC22380	CAGGGCCUCACUGUAUCGCCCA
4	POS CONTROL	has-miR-224	POS CONTROL	POS CONTROL	UCAAGUCACUAGUGGUUCCGUUUAG
5	MIMAT0019881	hsa-miR-4746-3p	22383	MC22383	AGCGGUGCUCCUGCGGGCCGA
6	MIMAT0019961	hsa-miR-4790-5p	22723	MC22723	AUCGCUUUACCAUUCAUGUU
7	MIMAT0019008	hsa-miR-3689d	22098	MC22098	GGGAGGUGUGAUCUCACACUCG
8	MIMAT0019024	hsa-miR-548al	21878	MC21878	AACGGCAAUGACUUUUGUACCA
9	MIMAT0018932	hsa-miR-378f	21788	MC21788	ACUGGACUUGGAGCCAGAAG
10	MIMAT0019215	hsa-miR-3177-5p	22218	MC22218	UGUGUACACACGUGCCAGGCGCU
11	MIMAT0003385	hsa-miR-363*	11353	MC11353	CGGGUGGAUCACGAUGCAAUUU
12	MIMAT0000226	hsa-miR-196a	10068	MC10068	UAGGUAGUUUCAUGUUGUUGGG
13	MIMAT0002807	hsa-miR-491-5p	11479	MC11479	AGUGGGGAACCCUUCCAUGAGG
14	MIMAT0018967	hsa-miR-4448	22317	MC22317	GGCUCCUUGGUCUAGGGGUA
15	MIMAT0003280	hsa-miR-612	11461	MC11461	GCUGGGCAGGGCUUCUGAGCUCCUU
16	MIMAT0003215	hsa-miR-552	11431	MC11431	AACAGGUGACUGGUUAGACAA
17	MIMAT0019796	hsa-miR-4700-5p	22042	MC22042	UCUGGGGAUGAGGACAGUGUGU
18	MIMAT0019728	hsa-miR-4660	22726	MC22726	UGCAGCUCUGGUGGAAAAUGGAG
19	MIMAT0019743	hsa-miR-4667-5p	21789	MC21789	ACUGGGGAGCAGAAGGAGAACC
20	MIMAT0019041	hsa-miR-4505	21694	MC21694	AGGCUGGGCUGGGACGGA
21	MIMAT0019766	hsa-miR-4681	21695	MC21695	AACGGGAAUGCAGGCUGUAUCU
22	MIMAT0000426	hsa-miR-132	10166	MC10166	UAACAGUCUACAGCCAUGGUCG
23	MIMAT0019029	hsa-miR-4494	22041	MC22041	CCAGACUGUGGCUGACCAGAGG
24	MIMAT0019046	hsa-miR-4509	22384	MC22384	ACUAAAGGAUAUAGAAGGUUUU
25	MIMAT0019782	hsa-miR-4691-3p	21517	MC21517	CCAGCCACGGACUGAGAGUGCAU
26	MIMAT0019786	hsa-miR-4694-5p	22219	MC22219	AGGUGUUAUCCUAUCCAUUUGC
27	MIMAT0019938	hsa-miR-4779	22573	MC22573	UAGGAGGGAAUAGUAAAAGCAG
28	MIMAT0001541	hsa-miR-449a	11127	MC11127	UGGCAGUGUAUUGUUAGCUGGU
29	MIMAT0019737	hsa-miR-4664-5p	21667	MC21667	UGGGGUGCCCACUCCGCAAGUU
30	MIMAT0000082	hsa-miR-26a	10249	MC10249	UUCAAGUAAUCCAGGAUAGGCU
31	MIMAT0000687	hsa-miR-299-3p	10448	MC10448	UAUGUGGGAUGGUAAACCGCUU
32	MIMAT0018992	hsa-miR-4465	22714	MC22714	CUCAAGUAGUCUGACCAGGGGA
33	MIMAT0019357	hsa-miR-3972	21943	MC21943	CUGCCAGCCCCGUUCCAGGGCA
34	MIMAT0019715	hsa-miR-4651	22425	MC22425	CGGGGUGGGUGAGGUCGGGC
35	MIMAT0002804	hsa-miR-488*	10241	MC10241	CCCAGAUAAUGGCACUCUCAA
36	MIMAT0003320	hsa-miR-650	11602	MC11602	AGGAGGCAGCGCUCUCAGGAC
37	MIMAT0004762	hsa-miR-486-3p	12986	MC12986	CGGGGCAGCUCAGUACAGGAU
38	MIMAT0019735	hsa-miR-4663	22073	MC22073	AGCUGAGCUCCAUGGACGUGCAGU
39	MIMAT0003309	hsa-miR-639	11645	MC11645	AUCGCUGCGGUUGCGAGCGCUGU
40	MIMAT0000073	hsa-miR-19a	10649	MC10649	UGUGCAAAUCUAUGCAAAACUGA
41	MIMAT0019846	hsa-miR-4726-3p	21639	MC21639	ACCCAGGUUCCCUCUGGCCGCA
42	MIMAT0018952	hsa-miR-4436a	22608	MC22608	GCAGGACAGGCAGAAGUGGAU
43	MIMAT0019844	hsa-miR-4725-3p	21937	MC21937	UGGGGAAGGCGUCAGUGUCGGG
44	MIMAT0004498	hsa-miR-25*	12401	MC12401	AGGCGGAGACUUGGGCAAUUG
45	MIMAT0019711	hsa-miR-4649-5p	21525	MC21525	UGGGCGAGGGGUGGGCUCUCAGAG
46	MIMAT0003219	hsa-miR-555	11635	MC11635	AGGGUAAGCUGAACCUCUGAU
47	MIMAT0019958	hsa-miR-4788	21698	MC21698	UUACGGACCAGCUAAGGGAGGC
48	MIMAT0004568	hsa-miR-221*	12613	MC12613	ACCUGGCAUACAAUGUAGAUUU
49	MIMAT0003250	hsa-miR-585	11554	MC11554	UGGGCGUAUCUGUAUGCUA
50	MIMAT0003335	hsa-miR-657	11637	MC11637	GGCAGGUUCUCACCCUCUCUAGG
51	MIMAT0019077	hsa-miR-1587	21951	MC21951	UUGGGCUGGGCUGGGUUGGG
52	MIMAT0000266	hsa-miR-205	11015	MC11015	UCCUUCAUUCCACCGGAGUCUG
53	MIMAT0019835	hsa-miR-4721	22385	MC22385	UGAGGGCUCCAGGUGACGGUGG
54	MIMAT0000462	hsa-miR-206	10409	MC10409	UGGAAUGUAAGGAAGUGUGUGG
55	MIMAT0000765	hsa-miR-335	10063	MC10063	UCAAGAGCAAUAACGAAAAAUGU
56	MIMAT0002850	hsa-miR-524-3p	10729	MC10729	GAAGGCGCUUCCCUUUGGAGU
57	MIMAT0002872	hsa-miR-501-5p	10266	MC10266	AAUCCUUUGUCCCUGGGUGAGA
58	MIMAT0002824	hsa-miR-498	11121	MC11121	UUUCAAGCCAGGGGGCGUUUUUC
59	MIMAT0019218	hsa-miR-3194-3p	22083	MC22083	AGCUCUGCUGCUCACUGGCAGU
60	MIMAT0004694	hsa-miR-342-5p	13066	MC13066	AGGGGUGCUAUCUGUGAUUGA
61	MIMAT0019830	hsa-miR-4717-3p	21882	MC21882	ACACAUGGGUGGCUGUGGCCU
62	MIMAT0004697	hsa-miR-151-5p	11537	MC11537	UCGAGGAGCUCACAGUCUAGU
63	MIMAT0004819	hsa-miR-671-3p	12333	MC12333	UCCGGUUCUCAGGGCUCCACC
64	MIMAT0000267	hsa-miR-210	10516	MC10516	CUGUGCGUGUGACAGCGGCUGA
65	MIMAT0001412	hsa-miR-18b	10466	MC10466	UAAGGUGCAUCUAGUGCAGUUAG
66	MIMAT0000077	hsa-miR-22	10203	MC10203	AAGCUGCCAGUUGAAGAACUGU
67	MIMAT0000736	hsa-miR-381	10242	MC10242	UAUACAAGGGCAAGCUCUCUGU
68	MIMAT0003269	hsa-miR-601	11482	MC11482	UGGUCUAGGAUUGUUGGAGGAG
69	MIMAT0000417	hsa-miR-15b	10904	MC10904	UAGCAGCACAUCAUGGUUUACA
70	MIMAT0003290	hsa-miR-621	11609	MC11609	GGCUAGCAACAGCGCUUACCU
71	MIMAT0002879	hsa-miR-507	10509	MC10509	UUUUGCACCUUUUGGAGUGAA
72	MIMAT0003225	hsa-miR-561	11540	MC11540	CAAAGUUUAAGAUCCUUGAAGU
73	MIMAT0003316	hsa-miR-646	11379	MC11379	AAGCAGCUGCCUCUGAGGC
74	MIMAT0019942	hsa-miR-4781-5p	22740	MC22740	UAGCGGGGAUUCCAAUAUUGG
75	MIMAT0003340	hsa-miR-542-5p	13010	MC13010	UCGGGGAUCAUCAUGUCACGAGA
76	MIMAT0004548	hsa-miR-129*	12962	MC12962	AAGCCCUUACCCCAAAAAGUAU
77	MIMAT0000440	hsa-miR-191	11717	MC11717	CAACGGAAUCCCAAAAGCAGCUG
78	MIMAT0003294	hsa-miR-625	12566	MC12566	AGGGGGAAAGUUCUAUAGUCC
79	MIMAT0003945	hsa-miR-765	11892	MC11892	UGGAGGAGAAGGAAGGUGAUG
80	MIMAT0003215	hsa-miR-552	11431	MC11431	AACAGGUGACUGGUUAGACAA
81	MIMAT0014996	hsa-miR-3131	16741	MC16741	UCGAGGACUGGUGGAAGGGCCUU
82	MIMAT0012735	hsa-miR-718	16440	MC16440	CUUCCGCCCCGCCGGGCGUCG
83	MIMAT0005950	hsa-miR-1306	13206	MC13206	ACGUUGGCUCUGGUGGUG
84	MIMAT0014995	hsa-miR-3130-5p	17993	MC17993	UACCCAGUCUCCGGUGCAGCC
85	MIMAT0018931	hsa-miR-4419a	20913	MC20913	UGAGGGAGGAGACUGCA
86	MIMAT0015045	hsa-miR-3170	18765	MC18765	CUGGGGUUCUGAGACAGACAGU
87	MIMAT0005940	hsa-miR-1282	13568	MC13568	UCGUUUGCCUUUUUCUGCUU
88	MIMAT0015039	hsa-miR-3165	18587	MC18587	AGGUGGAUGCAAUGUGACCUCA
89	MIMAT0005920	hsa-miR-1266	13349	MC13349	CCUCAGGGCUGUAGAACAGGGCU
90	MIMAT0005915	hsa-miR-1263	13293	MC13293	AUGGUACCCUGGCAUACUGAGU
91	MIMAT0016901	hsa-miR-4271	18627	MC18627	GGGGGAAGAAAAGGUGGGG
92	MIMAT0019859	hsa-miR-4734	20995	MC20995	GCUGCGGGCUGCGGUCAGGGCG
93	MIMAT0019873	hsa-miR-4742-3p	20629	MC20629	UCUGUAUUCUCCUUUGCCUGCAG
94	MIMAT0019981	hsa-miR-4802-5p	20718	MC20718	UAUGGAGGUUCUAGACCAUGUU
95	MIMAT0018931	hsa-miR-4419a	20913	MC20913	UGAGGGAGGAGACUGCA
96	MIMAT0019864	hsa-miR-3064-5p	20900	MC20900	UCUGGCUGUUGUGGUGUGCAA
97	MIMAT0018359	hsa-miR-3943	19620	MC19620	UAGCCCCCAGGCUUCACUUGGCG
98	MIMAT0019811	hsa-miR-4709-5p	20934	MC20934	ACAACAGUGACUUGCUCUCCAA
99	MIMAT0019062	hsa-miR-4524	21326	MC21326	AUAGCAGCAUGAACCUGUCUCA
100	MIMAT0019873	hsa-miR-4742-3p	20629	MC20629	UCUGUAUUCUCCUUUGCCUGCAG
